# Endothelium‐derived dopamine modulates EFS‐induced contractions of human umbilical vessels

**DOI:** 10.1002/prp2.612

**Published:** 2020-06-22

**Authors:** José Britto‐Júnior, David H. A. Pinheiro, Alberto F. O. Justo, Guilherme M. Figueiredo Murari, Rafael Campos, Fernanda V. Mariano, Valéria B. de Souza, André A. Schenka, Fabiola Z. Mónica, Edson Antunes, Gilberto De Nucci

**Affiliations:** ^1^ Department of Pharmacology Faculty of Medical Sciences State University of Campinas (UNICAMP) Campinas Brazil; ^2^ Superior Institute of Biomedical Sciences Ceará State University (UECE) Fortaleza Brazil; ^3^ Department of Pathology Faculty of Medical Science State University of Campinas (UNICAMP) Campinas Brazil; ^4^ Department of Pharmacology Institute of Biomedical Sciences University of São Paulo (USP) São Paulo Brazil; ^5^ Brazil University Fernadopolis Brazil

**Keywords:** dopamine, EFS, endothelium, haloperidol, human umbilical artery, human umbilical vein, idazoxan, L‐NAME, prazosin, tyrosine hydroxylase

## Abstract

Electrical field stimulation (EFS) induces contractions of both snake aorta and human umbilical cord vessels (HUCV) which were dependent on the presence of the endothelium. This study aimed to establish the nature of the mediator(s) responsible for EFS‐induced contractions in HUCV. Rings with or without endothelium from human umbilical artery (HUA) or vein (HUV) were mounted in organ bath chambers containing oxygenated, heated Krebs‐Henseleit's solution. Basal release of dopamine (DA), noradrenaline, and adrenaline was measured by LC‐MS‐MS. Cumulative concentration‐response curves were performed with dopamine in the absence and in the presence of L‐NAME or of dopamine antagonists. EFS studies were performed in the presence and absence of L‐NAME, the α‐adrenergic blockers prazosin and idazoxan, and the dopamine antagonists SCH‐23390 and haloperidol. Tyrosine hydroxylase (TH) and dopa‐decarboxylase (DDC) were studied by immunohistochemistry and fluorescence in situ hybridizations. Basal release of dopamine requires an intact endothelium in both HUA and HUV. TH and DDC are present only in the endothelium of both HUA and HUV as determined by immunohistochemistry. Dopamine induced contractions in HUA only in the presence of L‐NAME. Dopamine‐induced contractions in HUV were strongly potentiated by L‐NAME. The EFS‐induced contractions in both HUA and HUV were potentiated by L‐NAME and inhibited by the D2‐like receptor antagonist haloperidol. The α‐adrenergic antagonists prazosin and idazoxan and the D1‐like receptor antagonist SCH‐23390 had no effect on the EFS‐induced contractions of HUA and HUV. Endothelium‐derived dopamine is a major modulator of HUCV reactivity *in vitro*.

**Abbreviations**
DDC: dopa decarboxylase
EFS: electric field stimulation
HUA: human umbilical artery
HUCV: human umbilical cord vessels
HUV: human umbilical vein
LC‐MS‐MS: Liquid chromatography/tandem mass spectrometry
L‐NAME: Nω‐nitro‐L‐arginine‐methyl ester
TH: tyrosine hydroxylase

## 1 INTRODUCTION

Electrical field stimulation (EFS) is a technique in which an electrical stimulus is applied uniformly to an isolated tissue in short pulse width waves.^1^, ^2^ It may cause tissue contraction or relaxation depending on the mediators released.^1^, ^3^, ^4^ The proposed mechanism for EFS in isolated tissues is stimulation of intramural nerve endings.^5^ The sodium channel blocker tetrodotoxin is classically used to block neural stimulation.^6^ The EFS‐induced contractions of the aortae of the snakes *Crotalus durissus terrificus* and *Bothrops jararaca*,^7^ *Panterophis guttatus,* ^8^ and of the tortoise *Chelonoidis carbonaria* ^9^ are endothelium dependent and tetrodotoxin insensitive. The EFS‐induced contractions of human umbilical cord vessels (HUCV) are also dependent on the presence of the endothelium and are not affected by tetrodotoxin,^10^ the latter indicating lack of involvement of nerve terminals. Indeed, the umbilical cord has no innervation since no cholinergic or adrenergic nerve fibers have been identified by fluorescence.^11^ The nonselective alpha‐blocker phentolamine caused a significant inhibition of EFS‐induced HUCV contractions. However, this inhibition was observed only at high concentrations, indicating that it may be acting on a different population of receptors.^10^

In this study, the nature of the mediator was identified by liquid chromatography coupled to tandem mass spectrometry (LC‐MS‐MS), followed by a pharmacological characterization of the EFS‐induced contractions in both HUA and HUV* in vitro*.

## 2 METHODS

### 2.1 Study participants

Participants over the age of 18, undergoing the natural or cesarean delivery from Santa Casa de Vinhedo (Vinhedo‐SP) and Campinas Maternity Hospital (Campinas‐SP), were invited to take part in the study. The women were normotensive, and did not have preeclampsia, pregestational, or gestational diabetes mellitus and none were on regular medication. Written consent was obtained from those who agreed to participate. Umbilical cords from 67 volunteers aged 18‐47 years were used (from 18‐25, n = 23; from 26‐35, n = 25; and from 36‐47, n = 19 participants).

The investigation conformed to the principles outlined in the Declaration of Helsinki and the protocol was approved by the Ethics Committee of the Institute of Biomedical Sciences of the University of São Paulo—ICB/USP (protocol number 3.165.417).

### 2.2 Reagents

Adrenaline, noradrenaline, dopamine, adenosine 5′‐triphosphate (ATP), N^ω^‐Nitro‐L‐arginine methyl ester hydrochloride (L‐NAME), H‐[1,2,4]oxadiazolo[4,3‐a]quinoxalin‐1‐one (ODQ), and SCH‐23390 were purchased from Sigma‐Aldrich Chemicals Co. (St Louis, Missouri, USA). Haloperidol was bought from Nallin Farmácia e Manipulação Ltda (Itatiba‐SP, Brazil). Dopamine‐d3 hydrochloride, DL‐noradrenaline‐d6 hydrochloride, and adrenaline‐d6 hydrochloride were acquired from CDN Isotopes (Point Claire, Canada). Aluminum oxide and Harris’ Hematoxilin were purchased from Dinamica Quimica Contemporanea Ltda (Indaiatuba‐SP, Brazil). Sodium chloride (NaCl), potassium chloride (KCl), calcium chloride (CaCl_2_), magnesium sulfate (MgSO_4_), sodium bicarbonate (NaHCO_3_), potassium phosphate monobasic (KH_2_PO_4_), glucose, and entellan were bought from Merck KGaA (Darmstadt, Germany). Acetonitrile was obtained from J.T Baker (Phillipsburg, NJ, USA), and formic acid (HPLC grade) was purchased from Mallinckrodt (St Louis, MO, USA). Anti‐human calretinin (code: IS627) was purchased from DAKO (Agilent, USA). Anti‐tyrosine hydroxylase (code: ab76442), anti‐dopa decarboxylase (code: ab211535), and the secondary antibody (a goat anti‐chicken IgY [code: ab150169]) were purchased from Abcam (Cambridge, UK). The tertiary antibody, a rabbit anti‐goat IgG (code: AP106P), was purchased from Sigma/Merck (Darmstadt, Germany). NovoLink™ Max Polymer Detection System (code: RE7280‐k) and 3,3′ diaminobenzidine (DAB) were purchased from Leica Biosystems (UK). Citrate buffer, pepsin, 2XSSC, and DAPI (code: Z‐2028‐20) were bought from ZytoVision kit (Bremerhaven, Germany).

### 2.3 LC‐MS‐MS analysis

This study was carried out on human umbilical cord specimens obtained from 12 different placentae/patients. A segment of the umbilical cord—10‐20 cm from the insertion point in the placenta and 5 cm from the umbilicus—was removed by the obstetrician and placed in a container with Krebs‐Henseleit's solution. The Wharton's jelly was removed and the umbilical arteries (HUA) and the umbilical vein (HUV) were dissected. Vessel rings (two HUA and one HUV; 15 mm each ring; with or without endothelium) were incubated in 10 mL organ baths containing Krebs‐Henseleit's solution: (mM) NaCl (118), KCl (4.7), CaCl_2_ (2.5), MgSO_4_ (1.2), NaHCO_3_ (25), KH_2_PO_4_ (1.2), and glucose (5.6) gassed with a mixture of 95%O_2_: 5% CO_2_ (pH 7.4) at 37°C. After a period of 30 minutes, an aliquot of 2 mL of the supernatant was transferred to an Eppendorf tube and stored at −20°C until the time for analysis.

The dopamine, noradrenaline, and adrenaline concentrations in the Krebs‐Henseleit's solution were determined by liquid chromatography coupled to tandem mass spectrometry (LC‐MS/MS). The extraction procedure was similar to that described for extracting methyldopa from plasma.^12^ Briefly, 100 µL of the internal standards (dopamine‐d3, noradrenaline‐d6, and adrenaline‐d6 at 100 ng/mL) were added to the Krebs’ solution (2 mL) followed by 1.5 mL of deionized water. After vortexing for 10 seconds, 100 mg of Al_2_O_3_ was added and left for incubation for 20 minutes in an orbital agitator (Centrifuge 5810/ 5810 R). The tubes were then centrifuged at 2000 g for 4 minutes at 4°C and the supernatant discarded. The residue was washed 4 times with 2 mL of deionized water. After the final wash, 200 µL of a solution containing trifluoroacetic acid 0.1% in HCN/H2O (60/40l; v/v) was added. After vortexing for 40 seconds, the Eppendorf tubes were centrifuged for 2000 g for 5 minutes and the supernatant transferred to the vials for injection. The samples were analyzed by liquid chromatography coupled to a triple quadrupole mass spectrometer, LCMS‐8050 (Shimadzu).

The separation of catecholamines was performed on a 100 × 4.6 mm Lichrospher RP‐8 column (GL Sciences Inc) using acetonitrile/water (5/95, v/v) with 0.1% formic acid as mobile phase at a flow rate of 0.4 mL/min. The mass spectrometer operated in positive electrospray ionization mode (ES+) for catecholamine detection. The analyses were executed in selected Multiple Reaction Monitoring (MRM) detection mode. The transitions and retention times employed are described in the table below (Table 1).

**Table 1:** Mass spectrometry operating conditions

Analyte	MRM transition (m/z)	Q1 Prebias (V)	Collision energy	Q3 Prebias (V)	Retention time (min)
Dopamine	154.00 > 91.15	−12.00	−23.00	−18.00	3.12 ± 0.3
Noradrenaline	170.10 > 107.10	−12.00	−23.00	−18.00	2.97 ± 0.3
Adrenaline	184.20 > 107.00	−12.00	−23.00	−18.00	3.05 ± 0.3
Dopamine‐d6	157.00 > 93.00	−12.00	−23.00	−18.00	3.12 ± 0.3
Noradrenaline‐d3	176.10 > 158.10	−12.00	−23.00	−18.00	2.97 ± 0.3
Adrenaline‐d6	190.00 > 171.95	−12.00	−23.00	−18.00	3.05 ± 0.3

### 2.4 Immunohistochemistry

This assay was carried out on human umbilical cord specimens obtained from 8 different placentae/patients. The human umbilical cord samples were then fixed in 10% neutral buffered formalin and embedded in paraffin blocks. Each block was cut into serial 4‐µm‐thick sections which were mounted on positively charged slides prior to H&E, immunohistochemical, or fluorescence in situ hybridization (FISH) stainings.

Tissue sections were deparaffinized and rehydrated in graded alcohols to distilled water, and then they were incubated in 3% hydrogen peroxide for 10 minutes to block the endogenous peroxidase. Antigen retrieval was performed by heating slides in citrate buffer (10 mm, pH 6.0) at 95°C for 20 minutes (in a steamer set). Subsequently, they were left to cool down at room temperature and rinsed with PBS. Each slide was then incubated for 2 hours at room temperature with one of the primary antibodies. The primary antibodies used were (1) anti‐human calretinin (mouse monoclonal IgG1; clone: DAK‐calret 1; catalog code: IS627; immunogen: purified recombinant protein, expressed from the human malignant mesothelioma cell line [Mero‐41] calretinin encoding gene, in E. coli^1,2^; the epitope was not specified by the manufacturer; dilution: 1:200 in PBS; DAKO/Agilent, USA), (2) anti‐tyrosine hydroxylase (chicken polyclonal IgY; catalog code: ab76442; immunogen: two synthetic peptide/keyhole limpet hemocyanin [KLH] conjugates—these synthetic peptides corresponded to different regions of the Tyrosine Hydroxylase gene product, but were shared between the human [P07101] and mouse [P24529] sequences; predicted reactivity: mouse, rat, and human, according to the manufacturer; dilution 1:1500 in PBS, Abcam, Cambridge, UK), and (3) anti‐dopa decarboxylase (mouse monoclonal IgG1; clone: CL 2962; catalog code: ab211535; immunogen: recombinant fragment corresponding to Human DOPA Decarboxylase/DDC aa 114‐221; sequence: LETVMMDWLGKMLELPKAFLNEKAGEGGGVIQGSASEATLVALLAARTKVIHRLQAASPELTQAAIMEKLVAYSSDQAHSSVERAGLIGGVKLKAIPSDGNFAMRASA; database link: P20711; epitope: binds to an epitope located within the peptide sequence MDWLGKMLEL (aa 119‐128) as previously determined by the manufacturer using overlapping synthetic peptides; and dilution: 1:100 in PBS; Abcam, Cambridge, UK). Detection of tyrosine hydroxylase required the use of secondary and tertiary antibodies. The secondary antibody was a goat anti‐chicken IgY (catalog code: ab150169; dilution 1:500 in PBS, Abcam, Cambridge, UK). The tertiary antibody was a rabbit anti‐goat IgG (catalog code: AP106P, dilution 1:250 in PBS, Sigma/Merck, Germany). The slides were incubated for 1 hour at room temperature with the secondary antibody, followed by 1 hour incubation with the tertiary antibody. The detection system was the NovoLink™ Max Polymer Detection System (catalog code RE7280‐k, Leica Biosystems, UK), following the protocol described by the manufacturer. Thereafter, 3,3′ diaminobenzidine (DAB) was used as chromogen. Finally, the slides were dehydrated, counterstained with Harris´ hematoxylin and cover slipped in Entellan (Sigma/Merck). Negative controls consisted of the omission of the primary antibody and incubation with the primary antibody diluents (as well as with the secondary/tertiary antibodies, where applicable, and the detection system) and were performed in all immunohistochemistry assays (one negative control per section) to identify any background staining. All solutions (including primary, secondary, and tertiary antibody stocks) were prepared for a single use on the same day of the immunohistochemistry assay, and kept at 4ºC until use. All slides were examined and photomicrographed using a trinocular Eclipse 50i microscope (Nikon) coupled to a 10MP CMOS digital camera (AmScope, EUA). Positivity was assessed by an experienced MD, PhD pathologist (AAS), who was blind to the presence/absence of the primary antibody on the sample under examination (the observer did not know whether a test sample or an omission control was being assessed). Blinding was achieved by covering the slide labels with a removable occluding sticker.

For FISH analysis, sections from 5 randomly selected from the 8 human umbilical cords used for immunohistochemistry were deparaffinized with xylene and rehydrated in graded alcohols for 5 minutes each. Then, they were incubated in a 0.2 N HCl solution for 20 minutes, and subsequently treated with a citrate pH 6.0 buffer (ZytoVision kit, catalog code Z‐2028‐20, Germany) at 80ºC for 1 hour. After this, they were incubated with pepsin for 8 minutes at room temperature. The slides were washed with 2XSSC (ZytoVision kit, catalog code Z‐2028‐20, Germany), dehydrated in a sequence of ethanols (75%, 80%, and 100% ethanol for 2 minutes each), and then air dried. The slides were incubated with 100µL of the TH mRNA probe (at a concentration of 100 µM, in RNAse‐free water) for 10 minutes at 75ºC and overnight in a Dako Hybridizer (Dako, Denmark) at 37ºC. The TH mRNA probe sequence was as follows: 5′‐ AACCGCGGGGACATGATGGCCT‐3′ (RNA Tm = 77.8°C) (catalog code: VC00021, Sigma/Merck, Germany). The probe was labeled with fluorescein 6‐FAM in the 5’ region. The next day, the slides were placed in a UREA/0,1Xssc solution at 45°C for 30 minutes, and then, they were washed with a 2xSSC solution for 2 minutes. After this, the slides were dehydrated in 75%, 85%, and 100% ethanols for 2 minutes each, and air dried. Finally, the slides were mounted with 15 µL of a DAPI containing mounting medium (from the ZytoVision kit) and cover slipped (the cover slip being sealed with a Fixogum Rubber Cement, from Marabu, Germany). Negative controls consisted of the omission of the probe and were performed in all FISH assays (one negative control per section) to control for any significant autofluorescence. All FISH slides were examined and photomicrographed using a trinocular DM4000 B LED microscope (Leica Microsystems, Wetzlar, Germany) coupled to a 1.4 MP DFC 310 FX camera (Leica, Switzerland).

### 2.5 Pharmacological experiments

This study was carried out on human umbilical cord specimens obtained from 47 different placentae/patients. The Wharton's jelly was removed and the umbilical arteries (HUA) and the umbilical vein (HUV) were dissected. Vessels rings (3 mm) were suspended vertically between two metal hooks in 10 mL organ baths containing Krebs‐Henseleit's, gassed with a mixture of 95%O_2_:5% CO_2_ (pH 7.4) at 37°C, and coupled to isometric transducer. The initial smooth muscle tension was set at 10 mN.^10^ Tensioning force was recorded using a PowerLab 400 data acquisition system (Software Chart, version 7.0; ADInstruments, Colorado Springs, CO, USA).

Following a 90‐min stabilization period, the rings were precontracted with 5‐HT (1 µM), and the integrity of the endothelium in both HUA and HUV was evaluated by the addition of ATP to cause relaxation (10 µM).^10^ Cumulative concentration‐response curves to dopamine (10 nM to 3 mM) were performed in endothelium‐intact HUA and HUV rings in the absence and in the presence of the NO synthesis inhibitor L‐NAME (100 µM; for 60 minutes). The effect of D1‐like receptor antagonist SCH‐23390 (10 µM; for 30 minutes) and the D2‐like receptor antagonist haloperidol (10 µM; for 30 minutes) in dopamine‐induced contractions was investigated in endothelium‐intact vessels treated with L‐NAME.

The HUA and HUV rings were submitted to EFS at 60V for 30 seconds, at 8‐16 Hz in square‐wave pulses, 0.3 ms pulse width, and 0.1 ms delay, using a Grass S88 stimulator (Astro‐Medical, Industrial Park, RI, USA). Electrical field simulations were performed in the presence and absence of L‐NAME, and in the presence and absence of the dopamine D1‐like receptor antagonist SCH‐23390 (10 μM; for 30 minutes), of the dopamine D2‐like receptor antagonist haloperidol (10 μM; for 30 minutes), of the adrenergic alpha‐1 receptor antagonist prazosin (100 μM; for 30 minutes), and of the adrenergic alpha‐2 receptor antagonist idazoxan (100 μM; for 30 minutes). The effect of the antagonists was evaluated always in the presence of L‐NAME (100 μM).

### 2.6 Data analysis

Data are expressed as mean ± (SEM) of the number of experiments (n = or>5). Paired Student's *t* test was used and a *P*‐value < .05 was considered as significant. In the pharmacological experiments, the number of experiments is expressed as x/y, where x represents the number of umbilical vessels and y the number of rings employed in the experiment. For Emax analysis and pEC50, unpaired Student's *t* test was used and a *P*‐value of < .05 was considered as significant.

Nonlinear regression analysis to determine the pEC_50_ was carried out using GraphPad Prism (GraphPad Software, version 6.0, San Diego, CA, USA) with the constraint that Φ = 0. All concentration‐response data were evaluated for a fit to a logistics function in the form: E = Emax/([1 + (10^c^/10^x^)^n^] +Φ. The values of pEC_50_ data represent the mean ± SEM. Values of Emax were represented by mN.

## 3 RESULTS

### 3.1 Determination of amine concentrations by LC‐MS‐MS

**Figure 1:**
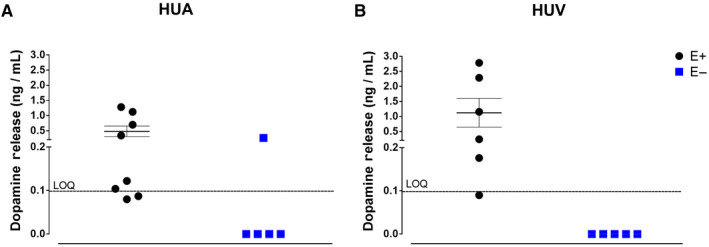
Panel A shows the basal release of dopamine in Krebs‐Henseleit´s solution after 30 minutes incubation with human umbilical artery endothelium‐intact rings (E+; n = 8) and with human umbilical artery endothelium‐denuded rings (E‐; n = 5). Panel B shows the basal release of dopamine in Krebs‐Henseleit´s solution after 30 minutes incubation with human umbilical vein endothelium‐intact rings (E+; n = 6) and with human umbilical vein endothelium‐denuded rings (E‐; n = 5). LOQ = limiti of quantitation

Dopamine, noradrenaline, and adrenaline calibration curves were linear for concentrations of 0.1‐10.0 ng/mL, with a correlation coefficient higher than 0.99. The limit of quantification was 0.1 ng/mL. The method was fully validated, and the results reported elsewhere.^13^ Only dopamine concentrations were above the limit of quantification, and were only observed in endothelium‐intact HUA and HUV (Figure 1A and 1B).

### 3.2 Umbilical cord vessels. Immunohistochemistry and fluorescence in situ hybridization (FISH) analysis

**Figure 2:**
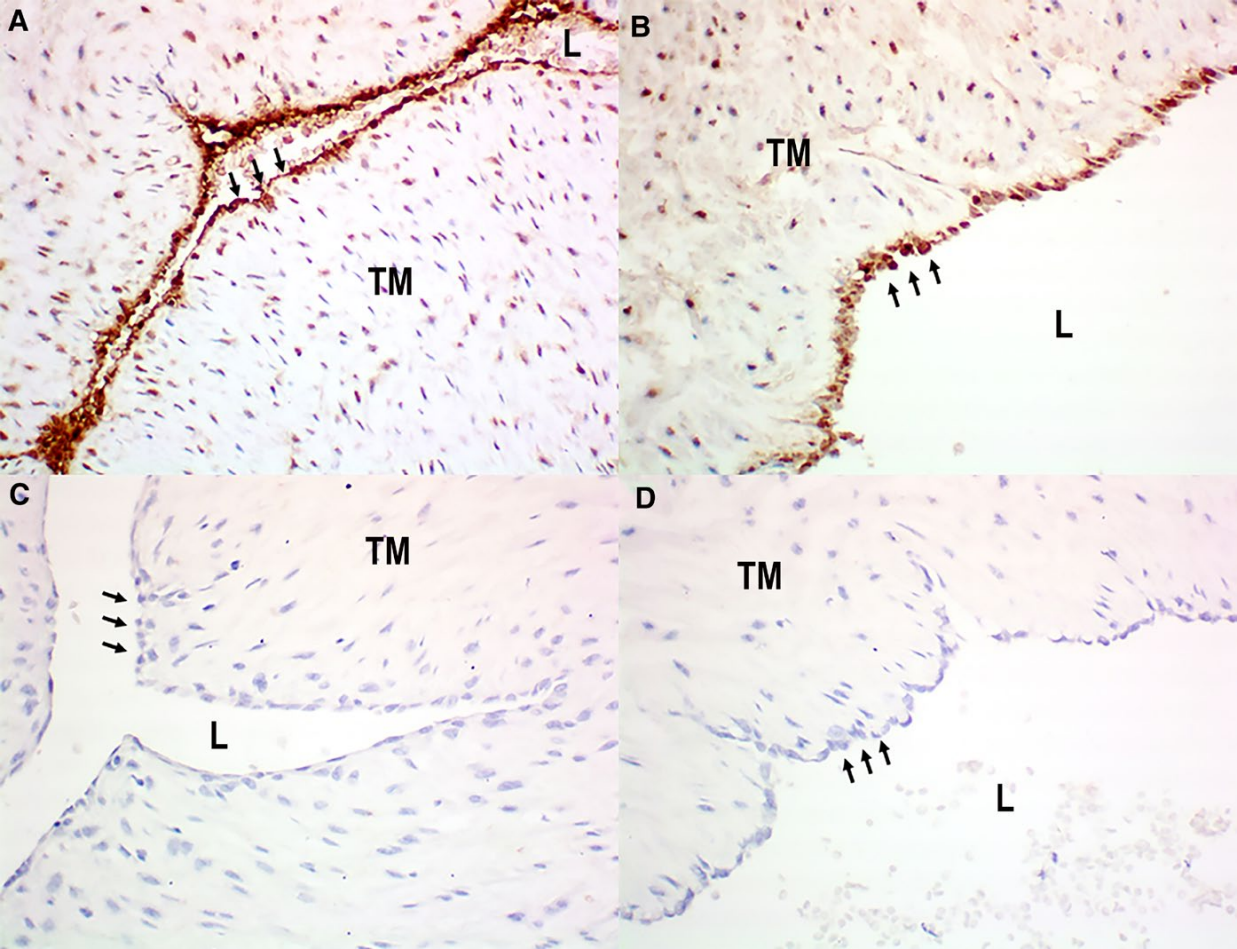
Detection of tyrosine hydroxylase by immunohistochemistry in human umbilical cords: positive endothelial staining in HUA (panel a [arrows]) and HUV (panel b [arrows]); negative control sections (omission of primary antibody) showing absence of positivity in both HUA (panel c [arrows]) and (panel d [arrows]) HUV endothelia. Immunoperoxidase (200X, original magnification). L = lumen; TM = Tunica Media

Tyrosine hydroxylase was detected by immunohistochemistry only in endothelial cells, in all samples of both HUA (n = 8; Figure 2A) and HUV (n = 8; Figure 2B). Negative controls were obtained by the omission of the primary antibody, as illustrated for HUA (n = 8; Figure 2C) and HUV (n* = *8, Figure 2D).

**Figure 3:**
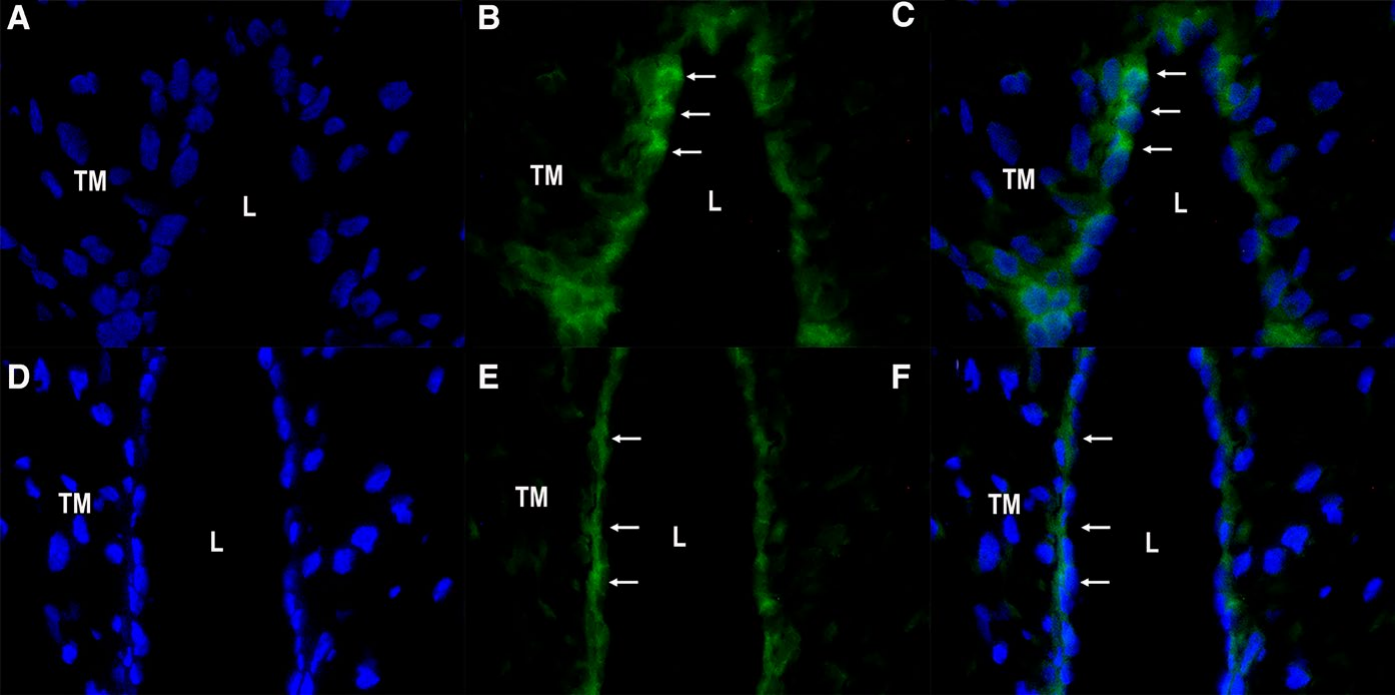
Detection of tyrosine hydroxylase mRNA by fluorescence in situ hybridization (FISH) in human umbilical cord artery (panel A‐C) and vein (panel D‐F); (panel A) HUA, DAPI staining in nuclei; (panel B) HUA, TH mRNA staining in the cytoplasm of endothelial cells (arrows); (panel C) HUA, overlay (DAPI + TH mRNA stainings [arrows]); (panel D) HUV, DAPI staining in nuclei; (panel E) HUV, TH mRNA staining in the cytoplasm of endothelial cells (arrows); (panel F) HUV, overlay (DAPI + TH mRNA stainings [arrows]). DAPI/FITC (400X, original magnification). L = lumen; TM = Tunica Media

Presence of tyrosine hydroxylase messenger RNA in HUA and HUV was assessed by fluorescence in situ hybridization analysis (FISH). Tyrosine hydroxylase mRNA was consistently detected in endothelial cells of all tested samples (n = 5), both in HUA (cytoplasmic green fluorescence in Figure 3B [cytoplasmic staining alone] and 3C [cytoplasmic and nuclear staining overlay]) and in HUV (cytoplasmic green fluorescence in Figure 3E [cytoplasmic staining alone] and 3F [cytoplasmic and nuclear staining overlay]). Endothelial nuclei are seen in blue (DAPI staining), both in HUA (Figure 3A [nuclear staining alone] and 3C [cytoplasmic and nuclear staining overlay]) and in HUV (Figure 3D [nuclear staining alone] and 3F [cytoplasmic and nuclear staining overlay]).

**Figure 4:**
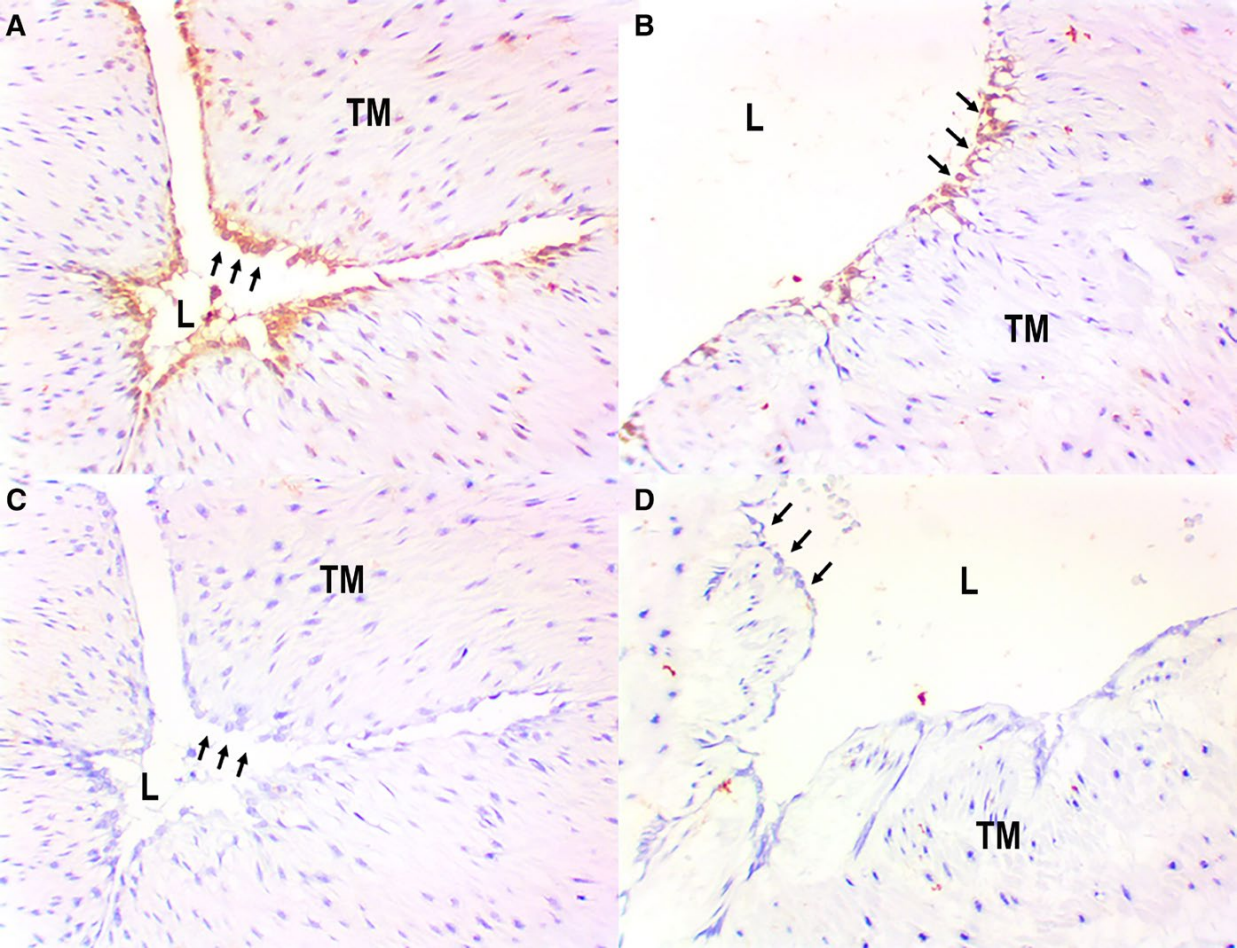
Detection of dopa decarboxylase by immunohistochemistry in human umbilical cords: positive endothelial staining in HUA (panel A [arrows]) and HUV (panel B [arrows]); negative control sections (omission of primary antibody) showing absence of positivity in both HUA (pane C [arrows]) and HUV (panel D [arrows]) endothelia. Immunoperoxidase (200X, original magnification). L = lumen; TM = Tunica Media

Dopa decarboxylase was detected by immunohistochemistry in the endothelia, in all samples of both HUA (n = 8; Figure 4A) and HUV (n = 8; Figure 4B). Negative controls were obtained by the omission of the primary antibody, as illustrated for HUA (n = 8; Figure 4C) and HUV (n = 8; Figure 4D). FISH could not be used to detect dopa decarboxylase mRNA because there were no commercially available probes at the time.

**Figure 5:**
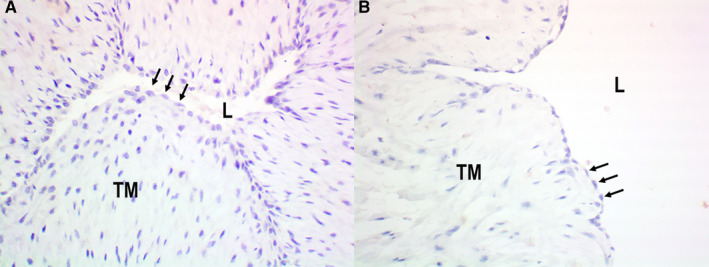
Detection of calretinin (CALRET) by immunohistochemistry in human umbilical cords: sections showing absence of positivity in both artery (panel A [arrows]) and vein (panel B [arrows]) endothelia. Calretinin is also negative in the tunica media (TM) of both vessels (panel A‐B). Immunoperoxidase (400X, original magnification). L = lumen; TM = Tunica Media

Using immunohistochemistry, we attempted to identify calretinin (a neural marker, commonly used to detect nerve fibers and neuronal cell bodies) in umbilical cord samples, with special attention to the vessel walls. Calretinin was not found in any samples of either HUA (n = 8; Figure 5A) or HUV (n = 8; Figure 5B), which indicates lack of neural tissue within the vessels walls (thus, ruling out a neural origin for the vessel‐derived catecholamines detected in the pharmacological assays).

### 3.3 Effect of L‐NAME

**Figure 6:**
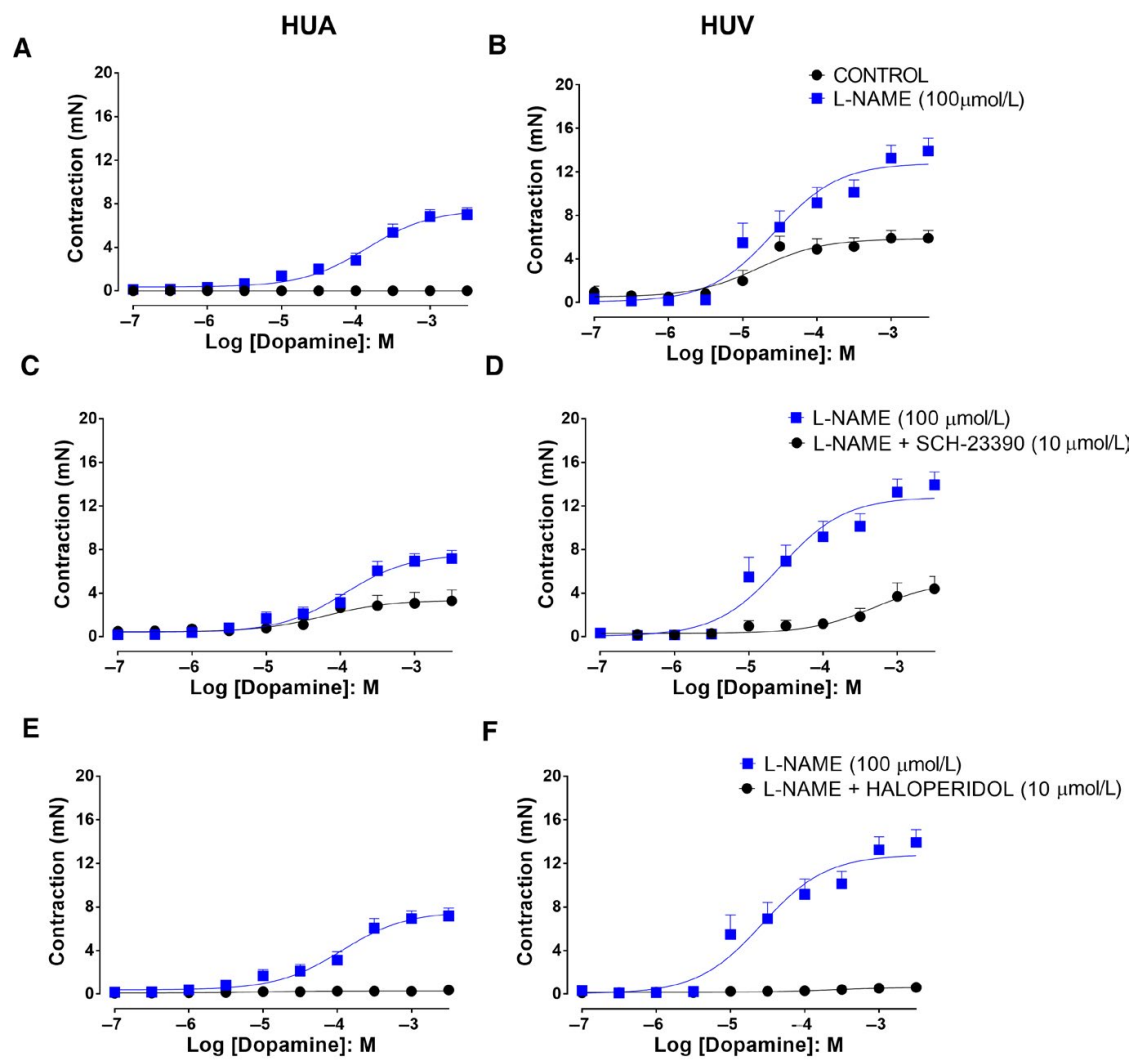
Dopamine concentration‐response curves in the absence and presence of L‐NAME in HUA rings (Panel A [control n = 6/6 and L‐NAME 100 µM n = 6/12]) and in HUV rings (Panel B [control n = 5/5 and L‐NAME 100 µM n = 5/10]). Effect of the D1‐like receptor antagonist SCH‐23390 on the dopamine concentration‐response curves in HUA rings (Panel C [L‐NAME 100 µM n = 5/10 and L‐NAME + SCH‐23390 10 μM n = 5/10]) and in HUV rings (Panel D [L‐NAME 100 µM n = 5/10 and L‐NAME + SCH‐23390 10 μM n = 5/10]). Effect of the D2‐like receptor antagonist haloperidol on the dopamine concentration‐response curves in HUA rings (Panel E [L‐NAME 100 µM n = 5/10 and L‐NAME + haloperidol 10 μM n = 5/10]) and in HUV rings (Panel F [L‐NAME 100 µM n = 5/10 and L‐NAME + haloperidol 10 μM n = 5/10]). In the six panels, there was a significant difference in the Emax (*P* < .05)

Dopamine alone induced contractions in L‐NAME (100 µM)‐treated HUA (Emax 7.5 ± 0.4 mN; pEC_50_ 3.8 ± 0.1 (n = 6/12; Figure 6A). Dopamine also induced contractions in ODQ (10 µM) pretreated HUA (Emax 6.9 ± 0.9 mN; pEC_50_ 4.1 ± 0.1; n = 5/10) and in endothelium‐denuded HUA (Emax 7.2 ± 1.0 mN; pEC_50_ 2.9 ± 0.1; n = 5/10). There was no significant difference in the Emax, but the pEC_50_ 2.9 of dopamine‐induced contractions in endothelium‐denuded HUA presented a significant right shift when compared to either L‐NAME‐ or ODQ‐treated HUA.

Dopamine caused concentration‐dependent contractions of HUV (Emax 5.9 ± 0.4 mN; pEC_50_ 4.8 ± 0.2 [n = 5/5]; Figure 6B), and those contractions were potentiated by previous incubation with L‐NAME (Emax 12.8 ± 0.7 mN; pEC_50_ 4.6 ± 0.1 [n = 5/10]; Figure 6B).

**Figure 7:**
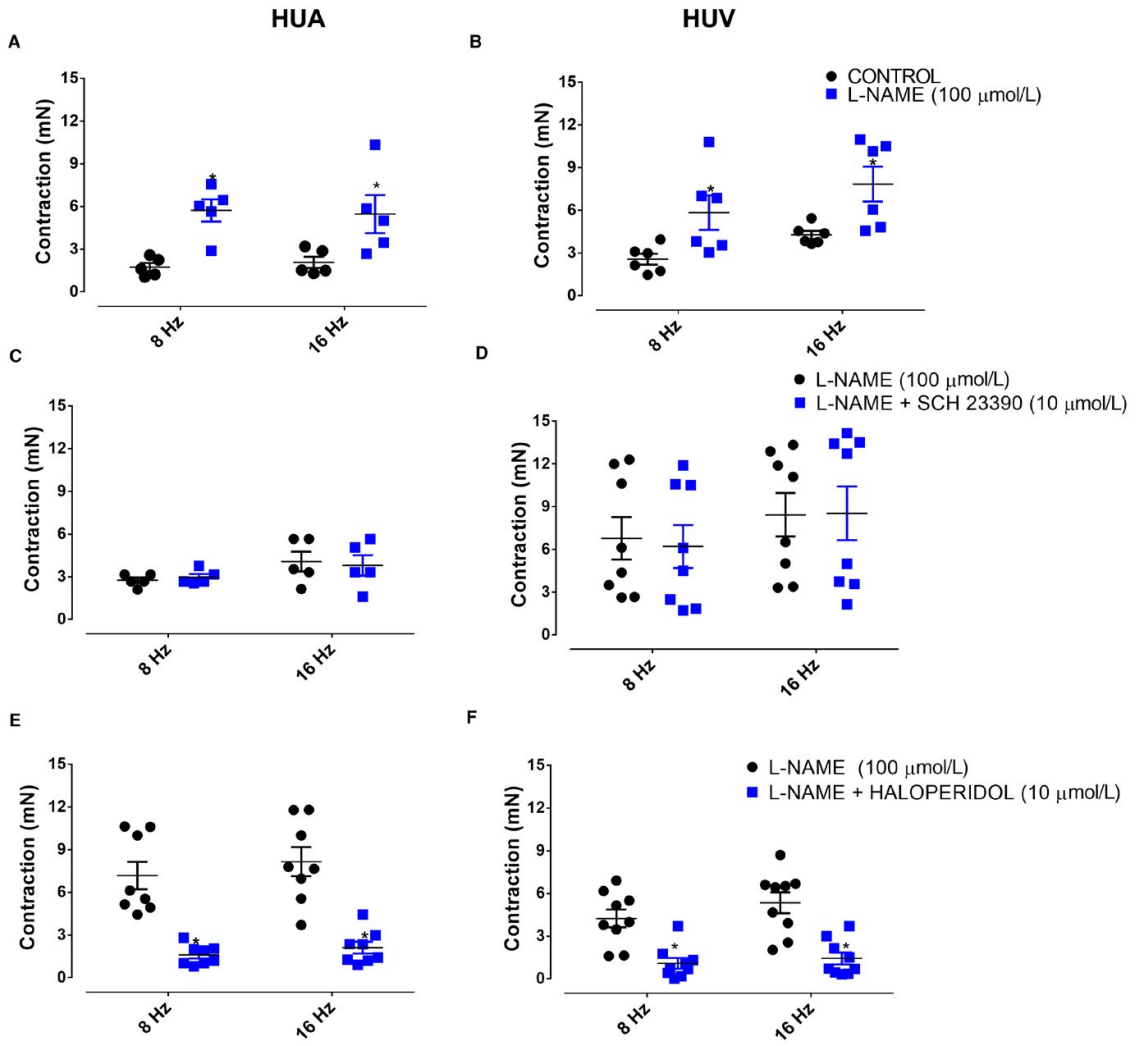
EFS caused a contraction in both HUA (panel A [control n = 5/5 and L‐NAME 100 µM n = 5/5]) and HUV rings (panel B [control n = 6/6 and L‐NAME 100 µM n = 6/6]). The response was significantly potentiated in both HUA and HUV by previous treatment with L‐NAME. The incubation with SCH‐23390 had no effect on the EFS‐induced contractions in either HUA (panel C [L‐NAME 100 µM n* = *5/5 and L‐NAME + SCH‐23390 10 μM n* = *5/5]) and HUV rings (panel D [L‐NAME 100 µM n* = *5/8 and L‐NAME + SCH‐23390 10 μM n* = *5/8]). The treatment with haloperidol caused significant reduction in EFS‐induced contractions in both HUA (panel E [L‐NAME 100 µM n* = *5/8 and L‐NAME + haloperidol 10 μM n* = *5/8]) and HUV rings (panel F [L‐NAME 100 µM n* = *5/9 and L‐NAME + haloperidol 10 μM n* = *5/9]). Data are expressed as mean ± SEM *P < .05. Vs control

Pretreatment with L‐NAME (100 µM) significantly increased the EFS (8 Hz and 16 Hz)‐induced contractions of both HUA (Figure 7A) and HUV (Figure 7B).

### 3.4 Effect of alpha‐adrenergic receptor antagonists

**Figure 8:**
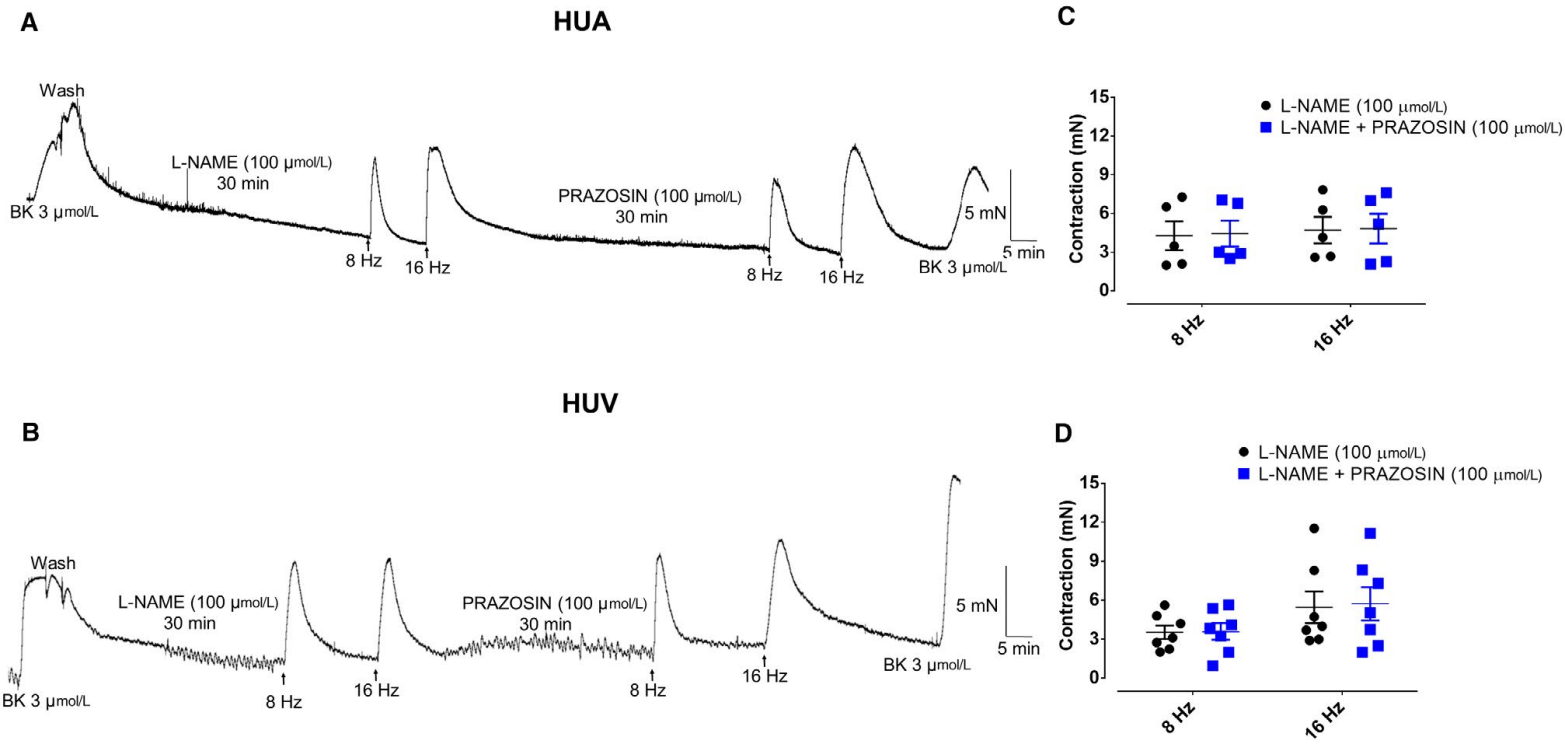
*8*The incubation of the alpha1‐adrenergic receptor antagonist prazosin has not affected the EFS‐induced contractions of either HUA (panel A [L‐NAME 100 µM n = 5/5] and [L‐NAME 100 µM + prazosin 100 µM n = 5/5]) or HUV rings (panel B [L‐NAME 100 µM n = 5/7] and [L‐NAME 100 µM + prazosin 100 µM n = 5/7]). Scatter plot shows the individual values and mean ± SEM of the EFS‐induced contractions in L‐NAME (100 µM) pretreated HUA (panel C; n = 5/5 for 8 Hz and 16 Hz) and HUV rings (panel D; n = 5/7 for 8 Hz and 16 Hz) in the absence and presence of prazosin

Incubation with prazosin (100 µM), a selective α_1_‐adrenoceptor antagonist, had no effect in the EFS‐induced contraction of the HUA (4.3 ± 1.1 and 4.5 ± 1.4 mN for 8 Hz; 4.7 ± 1.0 and 4.8 ± 1.2 mN for 16 Hz; n = 5/5, for control and prazosin pretreated vessels, respectively; Figure 8A,C). Similar results were obtained in HUV (3.5 ± 0.5 and 3.6 ± 0.6 mN for 8 Hz; 5.5 ± 1.2 and 5.7 ± 1.3 for 16 Hz; n = 5/7, for control and prazosin pretreated vessels, respectively; Figure 8B,D).

**Figure 9:**
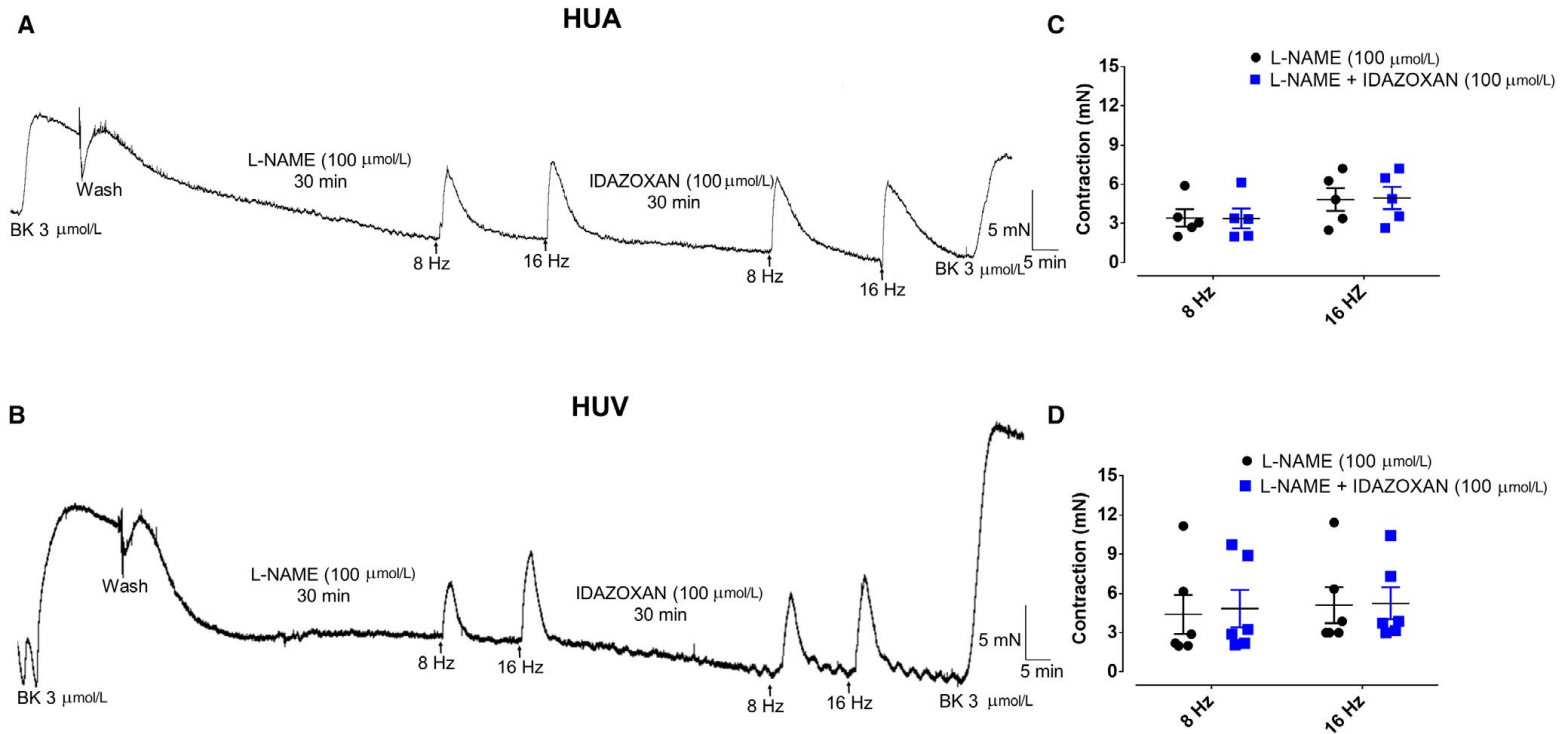
The incubation of the alpha2‐adrenergic receptor antagonist idazoxan has not affected the EFS‐induced contractions of either HUA (panel A [L‐NAME 100 µM n = 5/5] and [L‐NAME 100 µM + idazoxan 100 µM n = 5/5]) rings or HUV (panel B [L‐NAME 100 µM n = 5/6] and [L‐NAME 100 µM + idazoxan 100 µM n = 5/6]) rings. Scatter plot shows the individual values and mean ± SEM of the EFS‐induced contractions in L‐NAME (100 µM) pretreated HUA (panel C; n = 5/5 for 8 Hz and 16 Hz) and HUV (panel D; n = 5/6 for 8 Hz and 16 Hz) in the absence and presence of idazoxan

Incubation with idazoxan (100 µM), a selective α_2_‐adrenoceptor antagonist, had no effect in the EFS‐induced contraction of the HUA (3.4 ± 0.8 and 3.4 ± 1.0 mN for 8 Hz; 4.8 ± 1.2 and 4.9 ± 1.1 mN for 16 Hz; n = 5/5, for control and idazoxan pretreated vessels, respectively, Figure 9A,C). Similar results were obtained in HUV (4.4 ± 1.5 and 4.8 ± 1.4 mN for 8 Hz; 5.1 ± 1.4 and 5.2 ± 1.3 mN for 16 Hz; n = 5/6, for control and idazoxan pretreated vessels, respectively; Figure 9B,D).

### 3.5 Effect of dopamine receptor antagonists

In L‐NAME‐treated vessels, the dopamine D1‐like receptor antagonist SCH‐23390 (10 µM) caused reduction in dopamine‐induced contractions of HUA (Emax 7.6 ± 0.5 [n = 5/10] and 3.3 ± 0.5 mN [n = 5/10], without and with SCH‐23390, respectively; pEC_50_ 3.9 ± 0.1 [n = 5/10] and 4.2 ± 0.3 mN [n = 5/10], *P* < .05, without and with SCH‐23390, respectively; Figure 6C). Similar results were observed in HUV (Emax 12.8 ± 0.7 [n = 5/10] and 5.1 ± 0.9 mN [n = 5/10], without and with SCH‐23390, respectively; pEC_50_ 4.6 ± 0.1 [n = 5/10] and 3.3 ± 0.3 mN [n = 5/10], *P* < .05, without and with SCH‐23390, respectively; Figure 6D). The EFS (8 Hz and 16 Hz)‐induced contractions of both HUA (Figure 7C; n = 5/5) and HUV (Figure 7D; n = 5/8) were not affected by incubation with SCH‐23390 (10 µM).

In L‐NAME‐treated vessels, the dopamine D2‐like receptor antagonist haloperidol (10 µM) abolished the contraction dependent of dopamine HUA (Emax 7.6 ± 0.5 mN and pEC_50_ 3.9 ± 0.1 without haloperidol [n = 5/10]; Figure 6E) and HUV (Emax 12.8 ± 0.7 mN and pEC_50_ 4.6 ± 0.1 [n = 5/10], without haloperidol; Figure 6D). The Emax data there were significant difference *P* < .05. Incubation with haloperidol (10 µM) caused significant reduction in EFS‐induced contraction of the HUA (Figure 7E; n = 5/8) and HUV (Figure 7C; n = 5/9).

## 4 DISCUSSION

The endothelium of umbilical cord vessels is capable of releasing mediators capable of modulating the contractile activity induced by EFS.^9^ The results presented here clearly demonstrate, for the first time in human vessels, that HUA and HUV display a basal endothelium‐derived dopamine release, as identified by tandem mass spectrometry. Furthermore, the enzymes involved in dopamine synthesis, tyrosine hydroxylase, which is the enzyme responsible for the conversion of tyrosine into L‐dihydroxy‐phenylalanine (L‐DOPA)^14^ and dopa‐decarboxylase, also responsible for the conversion of L‐DOPA into dopamine,^15^ have been identified in the endothelial cells of both HUA and HUV by immunohistochemistry. The RNA messenger of tyrosine hydroxylase in the endothelial cells of both HUA and HUV was also characterized by fluorescence in situ hybridization. Cultured endothelial cells from bovine aorta^16^ and from rat mesenteric artery^17^ also express the enzymes involved in catecholamine synthesis. Thus, endothelium plays an obligatory role in dopamine release. In newborn Wistar rats has been demonstrated the non‐neuronal origin of dopamine. Indeed, chemical sympathectomy with 6‐hydroxydopamine caused a significant reduction in noradrenaline and adrenaline levels extracted from the aortae, while dopamine levels remained unaffected.^17^ The absence of neural/neuronal tissue within HUCV walls, as indicated by the absence of calretinin at these sites, supports the hypothesis of a non‐neuronal source for the vessel‐released dopamine we described.

Dopamine acts on selective receptors, belonging to the G protein–coupled receptor family. Five genes encoding DA receptors (DRs) have been identified. These receptors are divided into two subfamilies: the D1‐like receptor subtypes (D1R and D5R), coupled to Gs, activating adenylyl cyclase and the D2‐like subfamily (D2R, D3R, and D4R) coupled to Gi, inhibiting adenylyl cyclase.^18^ Dopaminergic receptors in vascular beds have been identified *in vitro* by radioligand‐receptor binding and autoradiographic techniques. The localization of dopamine‐1 (D_1_)^19^ and dopamine‐2 (D_2_) receptors has been assessed in smooth muscle tissue of rat cerebral, mesenteric and renal arteries.^19^ In cerebral, coronary, pulmonary, and mesenteric arteries of rabbits, dopamine D_1_ and D_2_ receptors have been localized in the endothelium.^20^ Immunohistochemical analysis has identified D_2_ and D_4_ subtypes in cerebral and mesenteric vascular bed and D_2_ and D_3_ receptors in renal vasculature, with the D_5_ subtype predominantly residing as a smooth muscle receptor in the vascular beds of rats.^21^ Similarly, in sections of HUA, the dopaminergic receptor D1 has been characterized by using the dopaminergic competitive antagonist SCH 23 390.^22^, ^23^ Dopamine is known to be able to cause endothelium‐dependent relaxation of rabbit pulmonary artery.^24^ Data assessing mRNA and/or protein expression of dopamine receptors in vessels seem to converge in showing D1‐like receptors expressed in endothelial cell.^25^ However, data on D1 signaling in endothelial cells are lacking.

The finding that dopamine could only contract HUA in the presence of the NO synthesis inhibitor L‐NAME indicates a major interaction of dopamine with NO on this vessel. Indeed, similar results were obtained with the heme‐site inhibitor of soluble guanylyl cyclase ODQ and in endothelium‐denuded vessel. It is likely that the dopamine released in the circulation by EC would cause NO‐dependent vasodilatation through the action on D1 receptors expressed by EC. Indeed, the hemodynamic effects of dopamine depend on the dose administered; with intravenous infusions ranging from 1 to 10 µg/kg/min, dopamine increased cardiac contractility,^26^ cardiac output,^27^ and renal blood flow^28^ in normal subjects. The heart rate did not change and the mean arterial blood pressure was either unchanged or slightly decreased. When higher infusion rates were administered, arterial pressure increased and heart rate decreased.^29^

The inhibition of EFS‐induced contractions by the D_2_‐like receptor antagonists haloperidol revealed another important modulator role of the endothelium‐derived dopamine, acting as a vasoconstrictor through the D2‐like receptor. The finding that SCH‐23390 also had some inhibitory effect on dopamine‐induced contractions of L‐NAME‐treated HUA and HUV was possibly due to the antagonistic effect of this compound on D2 receptors at higher concentration.^30^ Indeed, SCH‐23390 exhibits only 1/1,000th the potency of haloperidol as antagonist for the D2 receptor, which may explain the lack of effect observed in EFS‐induced contractions of HUCV. The role of D2‐like receptor as a modulator of vasoconstriction should not be restricted to the* in vitro* scenario. Domperidone and haloperidol applied as ophthalmic solutions in a rabbit ocular hypertensive model produced a marked increase in ocular blood flow.^31^ Administration of the selective D2 receptor agonist pramiprexole to healthy male volunteers caused increased systolic blood pressure in both supine and standing positions.^32^ In previous studies in the field of primary care, schizophrenic patients and nonschizophrenic patients treated with antipsychotics were strongly associated, after adjusted analysis, with a lesser presence of hypertension.^33^ This was particularly unexpected since patients affected by schizophrenia have an increased cardiovascular morbidity and mortality.^34^

The alpha_1_‐adrenergic receptor antagonist prazosin^35^ and the alpha_2_‐adrenergic receptor antagonist idazoxan^36^ had no effect on EFS‐induced contraction of HUCV, confirming that dopamine is the major catecholamine responsible for this phenomenon. The inhibition observed with phentolamine at higher concentration is possibly due to binding of phentolamine in D2‐like receptors, as previously suggested.^10^ Indeed, phentolamine at higher concentrations (>2 µM) displaces ^3^H‐haloperidol binding to dopamine receptors in calf brain membranes.^37^

The interaction between dopamine and NO should not be restricted to their pharmacological actions. Nitro‐catecholamines such as nitro‐dopamine, nitro‐noradrenaline, and nitro‐adrenaline have been found in rat brain.^38^ Thus, it is possible that endothelium‐derived dopamine may react with NO to form nitro‐dopamine, and nitro‐dopamine itself could be also an important mediator of cardiovascular reactivity. N‐arachidonoyl dopamine (NADA) is a member of the N‐acyl dopamine family; several lines of evidence identified NADA as an agonist of endo‐vanilloid receptors with similar potency of capsaicin.^39^ The finding that human vascular tissue displays basal release of endothelium‐derived dopamine warrants further investigation on whether or not these and other dopamine derivatives may have a modulatory role on the cardiovascular system.

What is the possible physiological role of endothelial‐derived dopamine? Although cardiac output is defined by the product of heart rate and systolic volume, it is known that the pumping function of the heart has a permissive role in the determination of cardiac output.^40^ Indeed, the cardiac output was largely unaffected by heart rate when subjects were electrically paced.^41^ The characteristics of the peripheral circulation such as capacitance and conductance/resistance play a major role in determining cardiac output. Most of the deductions in the role of sympathetic modulation of the circulation have been obtained by the use of either adrenergic agonists or antagonists, assuming that these mediators are coming from nerve terminals. The finding that human vascular tissue has basal release of dopamine should change this paradigm.

## CONFLICT OF INTEREST

The authors of this manuscript declare that they have no conflicts of interest.

Research data are not shared.

## AUTHOR CONTRIBUTIONS

**Conceptualization:** José Britto‐Júnior, Gilberto De Nucci. **Data curation:** Fabiola Z. Mónica, Edson Antunes, Gilberto De Nucci. **Formal analysis:** Gilberto De Nucci. **Funding acquisition:** Edson Antunes, Gilberto De Nucci. **Investigation:** José Britto‐Júnior, Gilberto De Nucci. **Methodology**: José Britto‐Júnior, David Halen Araújo Pinheiro, Alberto Fernando Oliveira Justo, Guilherme M. Figueiredo Murari, Rafael Campos, Fernanda Viviane Mariano, Valéria Barbosa de Souza, André Almeida Schenka, Edson Antunes, Fabiola Z. Mónica, Gilberto De Nucci. **Project administration:** Gilberto De Nucci. **Supervision:** Fabiola Z. Mónica, Edson Antunes. **Visualization:** Edson Antunes. **Writing – original draft:** José Britto‐Júnior, Gilberto De Nucci. **Writing – review & editing:** José Britto‐Júnior, Gilberto De Nucci.

### OPEN RESEARCH BADGES

This article has earned Open Data and Open Materials badges for making publicly available the digitally‐shareable data necessary to reproduce the reported results. The data and materials are available at DOI: 10.22541/au.158456487.79594821.
